# Automated Baseline-Correction and Signal-Detection Algorithms with Web-Based Implementation for Thermal Liquid Biopsy Data Analysis

**DOI:** 10.3390/cancers18010060

**Published:** 2025-12-24

**Authors:** Karl C. Reger, Gabriela Schneider, Keegan T. Line, Alagammai Kaliappan, Robert Buscaglia, Nichola C. Garbett

**Affiliations:** 1 Department of Mathematics and Statistics, Northern Arizona University, Flagstaff, AZ 86011, USA; kcr28@nau.edu (K.C.R.); ktl56@nau.edu (K.T.L.); 2 UofL Health—Brown Cancer Center, Louisville, KY 40202, USA; gabriela.schneider@louisville.edu (G.S.); alagammai.kaliappan@louisville.edu (A.K.); 3 Division of Medical Oncology and Hematology, Department of Medicine, University of Louisville, Louisville, KY 40202, USA

**Keywords:** baseline-correction algorithm, signal-detection algorithm, differential scanning calorimetry (DSC), thermal liquid biopsy (TLB), TLB profile, web application, blood plasma, urine

## Abstract

Background/Objectives: Differential scanning calorimetry (DSC) analysis of blood plasma, also known as thermal liquid biopsy (TLB), is a promising approach for disease detection and monitoring; however, its wider adoption in clinical settings has been hindered by labor-intensive data processing workflows, particularly baseline correction. Methods: We developed and tested two automated algorithms to address critical bottlenecks in TLB analysis: (1) a baseline-correction algorithm utilizing rolling-variance analysis for endpoint detection, and (2) a signal-detection algorithm that applies auto-regressive integrated moving average (ARIMA)-based stationarity testing to determine whether a profile contains interpretable thermal features. Both algorithms are implemented in ThermogramForge, an open-source R Shiny web application providing an end-to-end workflow for data upload, processing, and report generation. Results: The baseline-correction algorithm demonstrated excellent performance on plasma TLB data (characterized by high heat capacity), matching the quality of rigorous manual processing. However, its performance was less robust for low signal biofluids, such as urine, where weak thermal transitions reduce the reliability of baseline estimation. To address this, a complementary signal-detection algorithm was developed to screen for TLB profiles with discernable thermal transitions prior to baseline correction, enabling users to exclude non-informative data. The signal-detection algorithm achieved near-perfect classification accuracy for TLB profiles with well-defined thermal transitions and maintained a low false-positive rate of 3.1% for true noise profiles, with expected lower performance for borderline cases. The interactive review interface in ThermogramForge further supports quality control and expert refinement. Conclusions: The automated baseline-correction and signal-detection algorithms, together with their web-based implementation, substantially reduce analysis time while maintaining quality, supporting more efficient and reproducible TLB research.

## Simple Summary

Differential scanning calorimetry (DSC) analysis of blood plasma, also known as thermal liquid biopsy (TLB), generates thermal profiles that provide complementary diagnostic information to augment established clinical methods for disease detection and patient monitoring. However, broader clinical adoption has been limited by the need for manual, time-consuming data processing. To address this challenge, we developed two algorithms that automate key steps in the analysis workflow. The first performs baseline correction, an essential preprocessing step, achieving performance comparable to rigorous manual processing for plasma TLB data. Unfortunately, the algorithm is less reliable for TLB profiles with highly variable amplitudes. To overcome this limitation, we developed a second algorithm that detects profiles with insufficient signal, enabling users to focus on data containing meaningful information. Both tools are integrated into ThermogramForge, a free web application that guides users from data upload through interactive review to final report generation, substantially reducing processing time and improving accessibility.

## 1. Introduction

Each year, an estimated twelve million patients in the U.S. are misdiagnosed [1], leading to delays in appropriate treatment and substantial preventable morbidity and mortality. Commonly misdiagnosed conditions include cancers (particularly melanoma and breast cancer), myocardial infarction, Lyme disease, and autoimmune disorders such as lupus, which takes an average of six years to diagnose [2,3,4,5,6]. Although multiple factors contribute to this high rate of diagnostic error, a major underlying issue is the limited performance of existing diagnostic tools. In search of new technologies capable of providing more accurate and rapid clinical assessments, researchers have evaluated the potential of differential scanning calorimetry (DSC), an established analytical technique for measuring the thermal unfolding profiles (thermograms) of biomolecules, for disease detection and monitoring [7,8,9,10,11,12,13,14,15,16,17,18,19,20,21,22,23,24,25,26,27,28,29,30,31,32].

Over the past ~15 years, a growing body of research focused on DSC-based analysis of blood plasma and serum, also referred to as thermal liquid biopsy (TLB), has revealed unique and subtle variations in the thermodynamic properties of human plasma proteins that enable differentiation of clinical samples based on patient health status [12,13,14,15,16,22,23,24,25,26,31,32,33,34,35,36,37,38,39,40,41,42]. Unlike many common diagnostic assays that quantify a single biomarker, TLB provides a holistic “snapshot” of the composite behavior of all components of plasma that reflects changes in protein abundance, post-translational modifications, disease-associated biomarker interactions, and broader alterations in protein interaction networks [13,17,25,35,43,44,45,46,47,48,49]. Despite multiple promising reports, widespread clinical adoption of TLB-based diagnostics has been limited by low experimental throughput, the time- and labor-intensive nature of manual data processing, and the cost, non-portability, and operational demands of conventional DSC instrumentation, all of which reduce feasibility in clinical settings.

Although recent efforts by manufacturers and researchers have focused on the development of lower-cost, smaller, and more user-friendly DSC systems, including microfabricated sensor platforms [50,51,52,53], there remains a critical need for accessible tools to streamline and standardize TLB data analysis. Manual baseline correction—the process of modeling the underlying heat capacity baseline between native and unfolded protein states—is particularly time-consuming and represents a major bottleneck in TLB workflows [54]. Additionally, reliable discrimination between true thermal signals and noise-dominated profiles is essential for samples with variable protein concentrations, yet no automated methods have been available for this critical quality control step. This manuscript addresses these challenges through the development and implementation of two automated algorithms designed to improve the handling of TLB data: (1) a baseline-correction algorithm that automates baseline endpoint detection to objectively identify stable pre- and post-transition regions; spline-based baseline modeling with user-selectable endpoint strategies (innermost, midpoint, outermost) that enable reproducible baseline correction, and standardized profile interpolation to ensure consistency across datasets; and (2) an ARIMA-based signal-detection algorithm that distinguishes analyzable heat capacity signals from noise-dominated data, a critical feature for samples with variable protein concentrations (e.g., urine). These algorithms have been implemented in ThermogramForge, an open-source R Shiny web application that provides a complete workflow for TLB data analysis, from data upload and interactive baseline review to automated metric calculation using our previously published *tlbparam* package [55,56,57] and comprehensive report generation with full metadata capture for reproducibility of analyses. The performance of both the baseline-correction and signal-detection algorithms was systematically evaluated using TLB datasets for different types of biological samples (plasma, urine) and validated through comparison with manual adjudication performed by DSC experts. By automating the most labor-intensive analysis steps while preserving expert oversight capabilities, this framework substantially enhances the efficiency, reproducibility, and accessibility of TLB-based research and diagnostics.

## 2. Materials and Methods

Two automated algorithms were developed to improve the processing of TLB data: (a) a baseline-correction algorithm for automated subtraction of the pre- and post-transition baselines, and (b) a signal-detection algorithm for identifying TLB profiles with a discernable heat capacity signal. Both algorithms were developed using the R programming language (version 4.5.2; R Foundation for Statistical Computing, Vienna, Austria) and implemented as part of the *ThermogramBaseline* R package version 0.0.0.9900 [58]. Together with the *tlbparam* R package version 0.0.0.9000 [55,56,57], which calculates profile metrics (e.g., area, width, transition temperatures, maxima), these algorithms were integrated into ThermogramForge version 0.1.0 [59], an open-source R Shiny version 1.11.1.9001 [60] web application supporting end-to-end TLB data processing and analysis. ThermogramForge is hosted free-of-charge through Posit Connect (https://rbuscaglia-thermogramforge.share.connect.posit.cloud/). The algorithms and the ThermogramForge application rely on the *readxl* version 1.4.3 [61] and *tidyverse* version 2.0.0 [62] suite of R packages for easy implementation.

### 2.1. Baseline-Correction Algorithm

The baseline-correction algorithm involves three major steps: (1) identification of baseline endpoints within the pre- and post-transition regions; (2) construction of a baseline model using smoothing splines and linear interpolation between endpoints; and (3) baseline subtraction followed by interpolation to a uniform temperature grid for cross-sample comparability.

First, each TLB profile was trimmed to remove any large differences in heat capacity signal (from initial heater activation or post-transition protein aggregation/precipitation) and then divided into three segments: a pre-transition baseline (from the minimum recorded temperature to the lower baseline endpoint), a post-transition baseline (from the upper baseline endpoint to the maximum recorded temperature), and a central exclusion region (not included in baseline fitting). Next, baseline endpoints within the pre- and post-transition regions were identified using cross-validated spline fitting combined with a moving window analysis, with the window size typically matched to the instrument data acquisition rate (e.g., ~90 data points per minute). For each window position, the standard deviation of residuals between observed values and the spline-fit was calculated. The window was moved by one data point across each baseline region and the window yielding the lowest standard deviation was selected as the most stable baseline region. Finally, within this region, three candidate baseline endpoint positions were assessed: the outermost point (farthest from the exclusion region), the midpoint, and the innermost point (nearest to the exclusion region). The innermost point, which demonstrated the strongest agreement with manual endpoint selection, was used as the default setting. This procedure was applied independently to the pre- and post-transition regions.

Once baseline endpoints were selected, the baseline model was then generated by fitting smooth splines to the pre- and post-transition segments and connecting their endpoints with a linear segment across the exclusion region. After baseline subtraction, data were interpolated onto a uniform temperature grid (45–90 °C in 0.1 °C increments) using a smooth spline fit to enable direct cross-sample comparisons. The algorithm was implemented as the *auto.baseline()* function within the *ThermogramBaseline* R package, which includes user-adjustable parameters for exclusion region boundaries, baseline endpoint position (innermost, midpoint, outermost), temperature grid resolution, and window size. A companion function *multiple.auto.baseline()* supports batch processing.

### 2.2. Signal-Detection Algorithm

Signal detection was performed using a two-step time-series analysis procedure: (1) calculation of first-differenced TLB profile data, and (2) evaluation of the stationarity of the differenced data. The algorithm was implemented as the *signal.detection()* function within the *ThermogramBaseline* R package, which integrates with the *auto.arima()* function from the *forecast* package version 8.24.0.9000 [63] for stationarity testing.

The first difference of each TLB profile was calculated by subtracting each data point from the subsequent data point across the entire profile, a common approach for removing linear trends in time-series data such as instrument drift or other gradual shifts unrelated to the signal of interest. The differenced series was then evaluated for stationarity using the *auto.arima()* function, which fits an auto-regressive integrated moving-average (ARIMA) model to the input data and applies additional differencing steps if required to achieve stationarity. Profiles for which the *auto.arima()* function applied additional differencing to achieve stationarity were classified as containing a detectable signal. Conversely, profiles for which the first-differenced series was already stationary were classified as white noise (no discernable signal). After application of the *auto.arima()* function, the algorithm outputs a final binary classification result of either “Signal” or “No Signal”.

### 2.3. ThermogramForge Application

ThermogramForge provides a three-module workflow comprising (1) Data Overview, for uploading and managing raw TLB profiles; (2) Review Endpoints, for visualization and optional manual refinement of automatically detected baseline endpoints; and (3) Report Builder, for selection and export of TLB metrics calculated from processed profiles. The application supports CSV or Excel files up to 150 MB and automatically recognizes both single- and multi-sample data formats.

During batch processing, uploaded raw TLB profiles are analyzed using the *auto.baseline()* and *signal.detection()* functions from the *ThermogramBaseline* package, with user-configurable parameters such as window size, exclusion region boundaries, temperature grid resolution, and the baseline endpoint selection method to accommodate different sample types or analytical requirements. Following processing, quality control and optional manual refinement can be performed interactively in the Review Endpoints module. ThermogramForge implements interactive visualization using *plotly* version 4.10.4.9000 [64], enabling direct adjustment of baseline endpoints and real-time recalculation of the corrected profile. All manual edits are tracked by an integrated undo/redo system. Processed datasets can be exported as RDS (R data structure), CSV (wide-format), or Excel (multi-sheet, including data and metadata) files.

### 2.4. Sample Datasets and Testing Methodology

Development and testing of algorithms were performed using TLB data corrected for instrumental baselines and normalized to total protein concentration. Datasets consisted of 36 blood plasma samples (72 TLB profiles [44,55]) and 378 urine samples (749 TLB profiles; manuscript in preparation) spanning a broad range of protein concentrations and exhibiting variable amplitudes of the heat capacity signal. The experimental methods and instrumentation used for the collection of the TLB data used in this study are provided in the published references [44,55].

## 3. Results

### 3.1. Baseline Correction

#### 3.1.1. Development of the Baseline-Correction Algorithm

**Figure 1:**
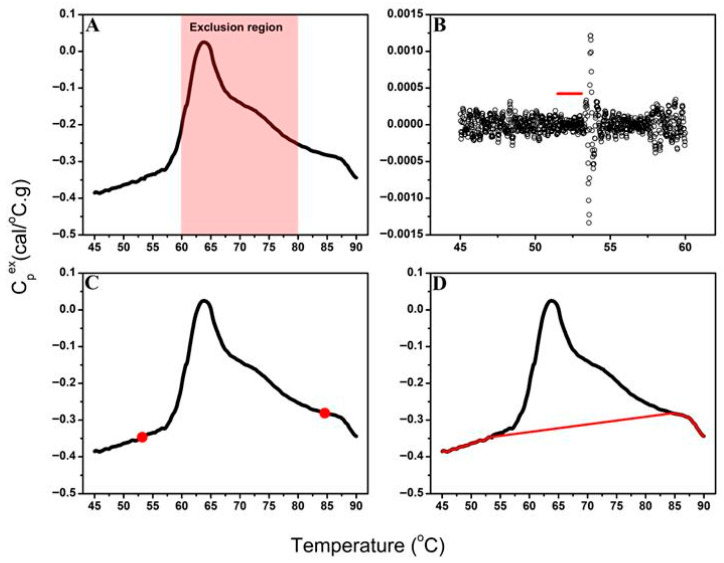
Overview of the automated baseline-correction algorithm workflow. (**A**) Example TLB profile showing the defined exclusion region (60–80 °C) excluded from baseline endpoint selection. (**B**) Variation in spline-fit residuals across the pre-transition segment; the red line indicates the most stable baseline segment identified by moving-window variance analysis. (**C**) TLB profile with automatically selected pre- and post-transition baseline endpoints (red circles) determined from the most stable baseline regions. (**D**) Final baseline model (red line) generated by connecting the pre- and post-transition spline fits with a linear segment across the exclusion region.

The automated baseline-correction algorithm accurately identified stable pre- and post-transition regions for plasma TLB profiles (Figure 1A,B). For plasma samples, the primary transition region is typically observed between 60 °C and 80 °C, with a smaller additional transition at ~50–55 °C [10,43].

Among the three endpoint-selection strategies (outermost, midpoint, and innermost), the innermost point (Figure 1C), positioned nearest to the exclusion region, provided the closest agreement with expert manual baseline selection upon empirical evaluation and was therefore implemented as the default algorithm setting. While midpoint selection represents an intuitive choice, systematic comparison against expert-corrected baselines revealed that the innermost strategy most consistently reproduced manual corrections across diverse sample types. The algorithm retains user flexibility to select alternative endpoint strategies (midpoint or outermost) to accommodate datasets where different positioning within the moving window may provide superior baseline estimates.

For the creation of the baseline model, a smooth spline fit was applied to the pre- and post-transition segments and linear regression was used to connect the baseline endpoints to span the exclusion region (Figure 1D). Although the algorithm can accommodate alternative baseline models (e.g., sigmoidal, cubic), selection of a linear fitting function for the sample transition region provided the most consistent and interpretable results for complex plasma profiles [65] and was implemented for development of the baseline model in this study.

#### 3.1.2. Performance of the Automated Baseline Algorithm Across Different Types of Biological Samples

Performance of the baseline-correction algorithm was evaluated using two distinct datasets. The first set consisted of duplicate TLB profiles for 36 plasma samples with diverse profile shapes. To account for the fibrinogen transition (~50–55 °C) and main protein unfolding transitions (~60–80 °C) in plasma samples, the exclusion region was set to 48–81 °C. Additional testing of alternative boundary ranges confirmed that this range provided optimal agreement with manual baseline correction, as it excluded all protein transitions while maximizing baseline regions.

**Figure 2:**
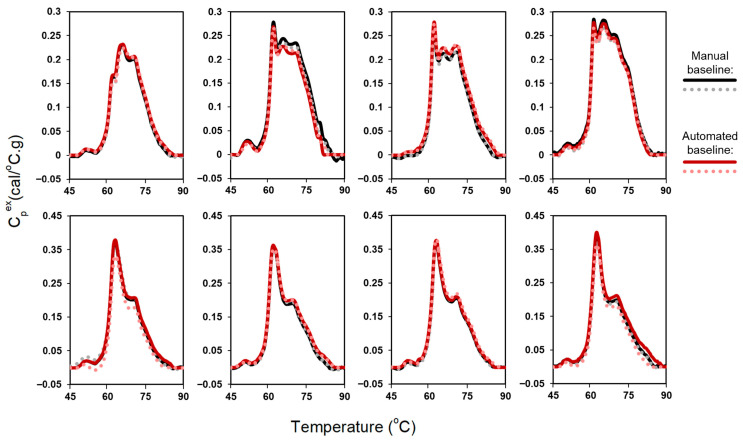
Comparison of automated and manual baseline correction for duplicate TLB profiles of 8 plasma samples.

As shown in Figure 2, automated baseline-corrected profiles exhibited strong agreement with manually corrected profiles. Following successful performance of the automated baseline algorithm using plasma data, we next assessed algorithm performance using urine TLB data (378 samples, 749 profiles). TLB analysis of urine samples is more challenging given the greater variability in protein concentration and, consequently, signal-to-noise ratio, associated with differences in patients’ kidney function. In addition, urine TLB profile features are less extensively characterized in our laboratory, contributing to greater uncertainty in profile interpretation. To accommodate the broader diversity of thermal features and minimize erroneous inclusion of transition-associated curvature in the baseline fit, the exclusion region was set to 60–80 °C. Overall, the automated baseline algorithm performed less robustly for urine samples than for plasma samples, likely reflecting the combined effects of signal-to-noise variability and greater heterogeneity in urine TLB profile shapes.

**Figure 3:**
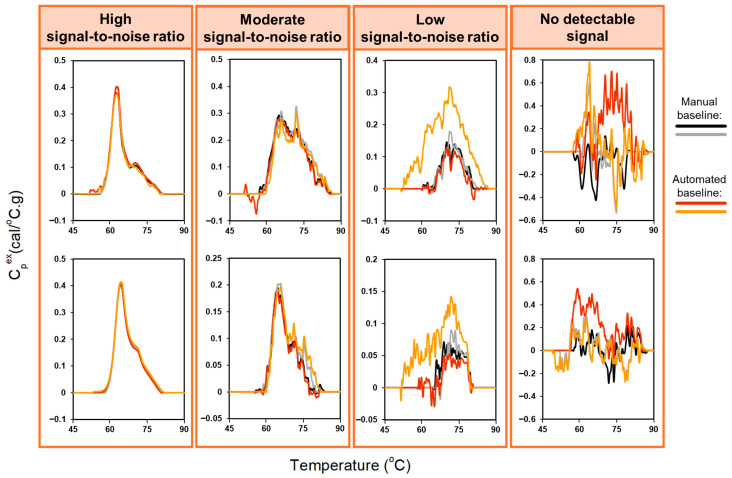
Comparison of automated and manual baseline correction for duplicate TLB profiles of 8 urine samples, with different amplitudes of the heat capacity signal: high signal-to-noise ratio, moderate signal-to-noise ratio, low signal-to-noise ratio, no detectable signal. The exclusion region used for the baseline-correction algorithm was set to 60–80 °C to accommodate the greater variability in thermal features observed for urine samples.

For urine profiles with high signal-to-noise ratios, automated and manual baseline corrections were highly consistent and comparable to the results obtained for plasma samples (Figure 3, left panel). In contrast, applying the automated baseline algorithm to profiles with moderate or low signal-to-noise ratios was more challenging (Figure 3, middle panels). When thermal transitions were well defined, both manual and automated baseline correction showed similar agreement. However, for profiles with weak or ambiguous thermal features, divergence increased. Profiles lacking discernable features (Figure 3, right panel) were particularly difficult to evaluate, even for DSC experts. The fundamental difficulty for low signal-to-noise profiles is that the algorithm cannot reliably detect clear peak departures from baseline, resulting in suboptimal baseline estimation. While adaptive exclusion regions would enable systematic evaluation across a grid of exclusion region parameters, this approach would substantially affect endpoint selection and yield different baseline approximations across profiles, compromising the standardization and reproducibility of the analysis. Moreover, adjusting the exclusion region is unlikely to overcome the inherent limitation of low signal TLB profiles, where the algorithm cannot reliably distinguish transitions from baseline noise. For cases where automated baseline detection performs poorly, the algorithm provides users with the capability to manually adjust the exclusion region parameters as an alternative approach. These challenges in identifying meaningful transitions motivated the development of an automated signal-detection algorithm to distinguish analyzable from non-analyzable TLB profiles prior to baseline fitting (Section 3.2).

### 3.2. Signal Detection

#### 3.2.1. Development of the Signal-Detection Algorithm

**Figure 4:**
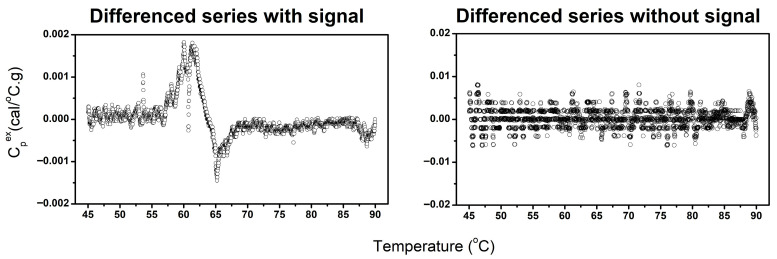
Examples of differenced TLB profiles classified as containing identifiable thermal transitions (**left**) and profiles lacking discernable thermal features (**right**).

Analysis of the first-differenced TLB profile data revealed clear distinctions between profiles containing meaningful thermal transitions and those dominated by noise. Our approach is based on the principle that DSC data typically exhibit linear or parabolic overall curve structures representing background drift, with biologically meaningful transitions appearing superimposed on this drift-like trend. By taking the first-difference of the TLB profile, the background drift is substantially diminished, leaving defined transitions in the differenced profile if true biological signal is present. To distinguish signal from noise, we employed the *auto.arima()* function to assess the stationary of the first-differenced TLB profiles. Stationarity is defined as a signal having a constant mean and variance with no trends or systematic patterns. The signal-detection approach operates on the principle that noise-dominated profiles become stationary after a single differencing step, whereas profiles containing biological signal require additional differencing to achieve stationarity because of the presence of defined transitions. Profiles whose first-differenced data appeared stationary were classified as “No signal”, indicating that no discernable thermal transitions were present, whereas profiles requiring additional differencing (i.e., non-stationary) were classified as “Signal”, corresponding to the presence of identifiable thermal features (Figure 4). It is important to note that we leveraged the automated stationarity detection capability of the *auto.arima()* function specifically for this classification task; the additional auto-regressive and moving-average components of the function are not currently utilized but could serve additional analytical purposes in future implementations. This approach provided an objective, quantitative criterion for determining whether a TLB profile contained interpretable signal before baseline correction.

#### 3.2.2. Performance of the Signal-Detection Algorithm Using Urine TLB Profiles

**Figure 5:**
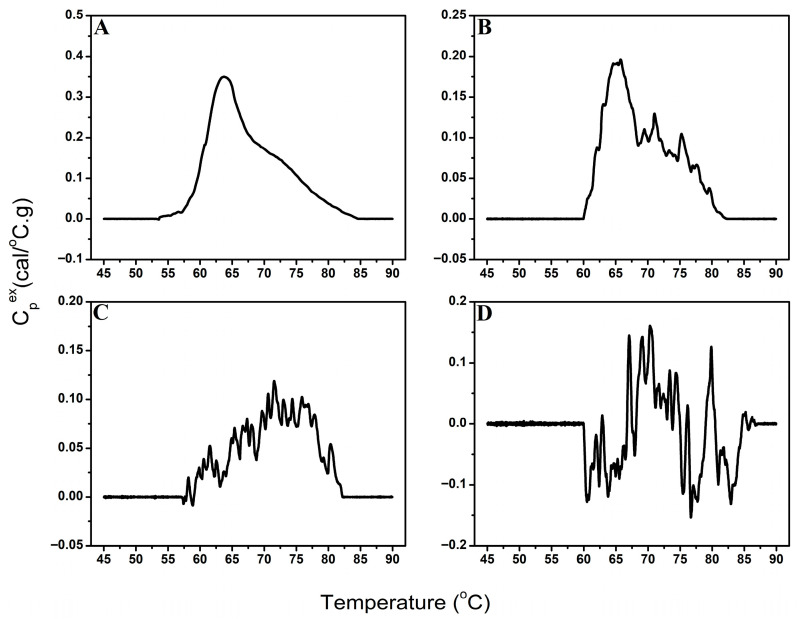
Examples of TLB profiles classified as high signal-to-noise (**A**), moderate signal-to-noise (**B**), low signal-to-noise (**C**), or no detectable signal (**D**).

The performance of the signal-detection algorithm was evaluated using 749 urine TLB profiles, using both baseline-corrected and raw profile data. Profiles were categorized into four groups (Figure 5) based on the strength of their thermal features—no discernable thermal transitions (no detectable signal), strong, well-defined thermal features (high signal-to-noise ratio), moderate, less-pronounced thermal features (moderate signal-to-noise ratio), and weak, minimally-detectable thermal features (low signal-to-noise ratio)—based on independent adjudication by three DSC experts using baseline-corrected data. Final classification was determined using a majority rule approach, with consensus classification requiring agreement of at least two reviewers. Algorithm performance was assessed using manual adjudication as the gold standard. Table 1 summarizes algorithm performance.

Across the 287 high signal-to-noise profiles, the algorithm showed excellent performance. Using raw TLB profiles, only one profile was incorrectly classified as having no signal, whereas two profiles were misclassified using baseline-corrected data, both likely affected by negative values (valleys) within the main transition signal. Manual adjudication showed similarly high consistency, with disagreement among the three reviewers for only one raw TLB profile and complete agreement for all baseline-corrected profiles.

Performance varied more substantially for TLB profiles with moderate signal-to-noise ratios. Using baseline-corrected data, the algorithm correctly identified 73 of 97 TLB profiles (24 misclassifications), whereas accuracy improved markedly when raw data were evaluated, with only four misclassifications. Manual adjudication remained highly consistent, with disagreement for only a single profile, independent of data type.

Classification of the remaining TLB profiles, those with low signal-to-noise or no signal, proved challenging for both automated and manual approaches. Among the 172 TLB profiles assigned to the low signal-to-noise group, the algorithm correctly identified only 9 profiles using baseline-corrected data. In contrast, similar to TLB profiles with moderate signal-to-noise ratios, performance improved substantially when raw TLB profiles were used, with 50% of profiles correctly classified. Reviewer agreement was also reduced in this group, particularly for raw TLB data (32 disagreements) compared with baseline-corrected data (7 disagreements).

For TLB profiles designated as “no signal” by manual adjudication, the algorithm performed well with baseline-corrected data, misclassifying only 6 of 193 profiles, but was less accurate using raw data (39 misclassifications out of 193 profiles). Reviewer disagreement was modest and again decreases slightly using baseline-corrected data (discordance for 13 profiles using raw TLB data and 7 after baseline correction).

Overall, manual adjudication was most consistent when performed using baseline-corrected TLB profiles, whereas the automated signal-detection algorithm performed best using raw data, particularly for profiles with moderate or low signal-to-noise ratios. This likely reflects artifacts introduced during baseline correction of lower signal profiles, where inaccurate endpoint detection, rather than spline fitting itself, can lead to substantial distortions in the spline estimation and subsequent baseline subtraction. These baseline-correction anomalies can obscure or distort subtle transition features and increase the apparent stationarity of the profiles, leading the algorithm to misclassify them as noise. For high signal-to-noise TLB profiles, endpoint detection is more reliable; consequently, both manual and automated signal-detection methods performed well regardless of whether raw or baseline-corrected data were used. Raw TLB data thus provide the most reliable input for automated signal-detection, particularly for profiles with lower signal-to-noise ratios, where baseline-correction artifacts arising from inaccurate endpoint identification may obscure subtle transitions and reduce classification accuracy. This sensitivity to endpoint placement underscores the importance of profile-by-profile review and motivated the inclusion of manual adjustment capabilities in the baseline-correction algorithm, allowing users to refine endpoint detection when encountering suboptimal performance of the automated algorithm.

### 3.3. Integration Within ThermogramForge

**Figure 6:**
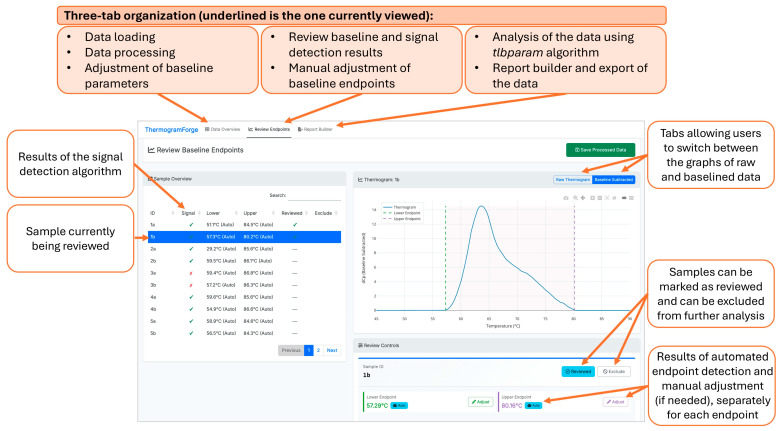
ThermogramForge R Shiny online dashboard with Review Endpoints panel on display. Dashboard panel outputs the algorithm results in an easy to review interface. Graphs of raw and processed TLB profiles are available and can be adjusted manually to refine profiles. A summary overview of all samples is given, highlighting signal/noise, endpoint detection, and allowing for review/exclude designations as work is completed.

Following testing of the *ThermogramBaseline* package in R, both the baseline-correction and signal-detection workflows were reimplemented using R Shiny and integrated into ThermogramForge, a web-based platform providing expanded analytical functionality (Figure 6). The software automates batch processing, baseline endpoint detection, and signal classification while supporting manual refinement through an intuitive graphical interface.

All parameter settings are stored per sample and may be propagated across analytical batches. ThermogramForge datasets saved in RDS format capture the full application state (manual endpoint adjustments, review status flags, and sample exclusion designations), enabling exact session restoration for verification, continued analysis, or independent review by collaborators. Excel exports include a dedicated metadata sheet recording timestamps, source dataset name, sample counts, and a list of evaluated metrics, with future implementation planned to include final baseline endpoint values and manual adjustment indicators. This approach strikes a balance between automated efficiency and expert quality control while ensuring reproducibility through comprehensive metadata capture. Additionally, integration of the *tlbparam* package enables automated extraction of quantitative TLB metrics following baseline correction. The software executes the *ThermogramBaseline* and *tlbparam* packages sequentially, ensuring that only TLB profiles containing interpretable thermal transitions are identified for baseline correction and downstream analysis. This framework supports high-throughput workflows while maintaining expert oversight through user-adjustable parameters and interactive visualization tools. Table S1 provides TLB profile data for six samples that allow users to test ThermogramForge and become familiar with its functionality.

## 4. Discussion

Baseline correction is a necessary but inherently uncertain step in DSC analysis and a major source of systematic error [54,66,67,68,69]. Because the baseline is not uniquely defined, small changes in its slope or curvature can substantially affect extracted thermodynamic parameters, particularly calorimetric enthalpy and transition width [54,66,67,68]. These effects are amplified for broad or overlapping transitions and for complex samples, where baseline selection often relies on subjective choices [65,68]. Despite the critical importance of baseline correction in DSC workflows, no standardized automated methods have been available for TLB data analysis, leaving researchers dependent on time-consuming manual processing that introduces operator-dependent variability and limits analytical throughput. Moreover, no automated approaches have existed for the essential quality control step of distinguishing TLB profiles containing interpretable thermal signals from noise-dominated data, a challenge particularly acute for biofluids with variable protein concentrations. To address these limitations, we developed novel automated algorithms for both baseline correction and signal detection that apply objective, reproducible criteria across datasets. The baseline-correction algorithm introduces several key innovations: a rolling variance method for automated endpoint detection that dynamically adapts to local baseline characteristics; spline-based baseline modeling with user-selectable endpoint strategies (innermost, midpoint, outermost) to accommodate diverse profile shapes; and standardized profile interpolation to ensure consistency. This approach replaces subjective manual baseline selection with a systematic, reproducible framework while retaining flexibility for expert refinement when needed. Complementing this, we developed an ARIMA-based signal-detection algorithm that automatically classifies TLB profiles as containing discernable thermal transitions or representing noise-dominated data. This algorithm leverages stationarity assessment of first-differenced TLB data to objectively distinguish biological signal from noise prior to baseline correction, enabling efficient pre-screening and ensuring that downstream analyses focus on interpretable profiles.

The automated baseline-correction algorithm performed robustly for plasma samples, yielding results comparable to manual correction while substantially reducing processing time. By rapidly identifying stable pre- and post-transition regions and applying consistent criteria across all TLB profiles, the algorithm eliminates the need for labor-intensive manual adjustment. When desired, users may further refine baseline endpoints through the *ThermogramBaseline* R package or within the ThermogramForge application. The rolling variance method for endpoint detection enabled rapid and objective identification of stable baseline segments, even for datasets with substantial variability in TLB profile shape. Importantly, the automated procedure enabled high-throughput processing, enabling baseline correction to be performed in a fraction of the time required for manual review. This gain in speed, combined with consistent application of baseline detection criteria, minimizes operator-dependent variability and enhances reproducibility across large datasets. Although automated baseline correction was comparable to manual processing, the primary advantage of the algorithm lies in its ability to process large TLB datasets quickly and reproducibly, thereby facilitating efficient analysis in studies involving hundreds of samples.

Performance of the baseline algorithm was less robust for urine samples, reflecting the broader challenge of developing universally applicable automated methods for highly heterogeneous biological samples. Compared with plasma, urine exhibits substantially greater variability in protein concentration and TLB profile shape, both of which depend on kidney function, hydration status, and other physiological factors. This variability hindered the algorithm’s ability to consistently identify appropriate baseline endpoints, particularly for TLB profiles with moderate or low signal-to-noise ratios. While adaptive exclusion regions would enable systematic evaluation across a range of exclusion parameters, this approach would substantially affect endpoint selection and yield different baseline approximations across profiles, compromising the standardization and reproducibility essential for clinical application. Moreover, adjusting the exclusion region parameters is unlikely to overcome the fundamental limitation of low signal-to-noise profiles, where the algorithm cannot reliably distinguish transition signals from baseline noise. To mitigate this challenge, we developed a complementary signal-detection algorithm designed to determine whether a TLB profile contains a detectable thermal signature prior to baseline correction. This step enables users to identify and exclude noise-dominated or non-informative data, thereby focusing downstream analyses on profiles with interpretable thermal features. The ARIMA-based signal-detection algorithm demonstrated strong performance for well-defined profiles, achieving high accuracy for high signal-to-noise data and a low false positive rate (3.1%) for true noise profiles. In contrast, classification of moderate or low signal-to-noise profiles was substantially more difficult. When applied to baseline-corrected data, correct classification was achieved for only 75.3% and 5.2% of moderate and low signal-to-noise profiles, respectively. Notably, performance improved markedly when raw TLB profile data were analyzed (95.5% and 50%, respectively), suggesting that automated baseline-correction artifacts arising from inaccurate endpoint identification can lead to substantial changes in the spline estimation and subsequent baseline subtraction that may distort or suppress subtle transitions in high noise profiles. The difficulty of signal detection for lower signal profiles was further illustrated by manual adjudication, where even expert reviewers disagreed on the classification of 32 raw profiles. Together, these findings underscore the inherent complexity of signal identification in urine TLB data and emphasize the need for cautious interpretation of profiles with subtle thermal features.

During algorithm development, we evaluated several alternative approaches. For baseline endpoint detection, methods based on absolute residual thresholds or fixed percentile cutoffs performed poorly, as they were highly sensitive to signal amplitude and exhibited limited robustness across diverse sample types. In contrast, the rolling variance method provided a more adaptive and reliable strategy, adjusting dynamically to local baseline characteristics while maintaining consistent performance across TLB profiles of varying amplitudes. For signal-detection, we considered spectral analysis, wavelet decomposition, and machine learning classifiers. Ultimately, the ARIMA-based approach was selected based on its strong theoretical foundation, interpretability, and robust performance without the need for extensive training data, making it well-suited for high-throughput analyses of heterogeneous biological samples.

The implementation of both algorithms within the ThermogramForge R Shiny application offers several advantages over standalone processing, particularly for users without extensive experience with the R environment. The interactive review interface enables quality control and manual refinement, allowing for visualization of both raw and baseline-corrected TLB profiles, while the undo/redo functionality ensures that all manual refinements are reversible and fully documented. The application supports data export in multiple formats with complete metadata documentation. The ThermogramForge architecture was designed to embody a core principle: accelerating throughput through automated processing while ensuring reproducibility and traceability through comprehensive metadata capture. Integration with the *tlbparam* package enables automated calculation of metrics, providing a complete end-to-end workflow from raw data to final quantitative results. This unified approach eliminates the need for data transfer between multiple software tools, reducing the risk of errors and improving overall efficiency. Currently, the use of the *tlbparam* package within ThermogramForge is limited to TLB profiles processed with the automated baseline algorithm; a future update will expand functionality to enable independent operation of the two packages.

Current limitations of the automated workflow include reduced performance for borderline or low-signal profiles, which can affect the reliability of baseline correction. Future development could address these challenges by incorporating confidence scores or probability estimates for signal classification, enabling more nuanced interpretation of profiles with weak thermal features. Implementation of sample-type-specific parameter sets (e.g., optimized window sizes or exclusion regions for different biofluids) could further enhance algorithm performance across diverse applications. Additional improvements, such as integration of batch comparison and statistical testing modules, would further support comparative studies and more comprehensive data analysis.

Overall, the development of automated algorithms for each step of TLB data processing workflow represents an important advance toward broader research and clinical utility of TLB. By automating the most labor-intensive analysis steps while preserving the ability for expert review and correction, the *ThermogramBaseline* package enables the analysis of larger sample cohorts with greater efficiency and consistency. Implementation of these algorithms within the open-source ThermogramForge R Shiny application provides a comprehensive platform that combines automated processing with expert oversight. The interactive review interface, flexible data management features, comprehensive metadata capture, and integration with metric calculation supports a complete workflow from raw data to quantitative results, facilitating more efficient and reproducible TLB research.

## 5. Conclusions

This work presents a robust computational framework for automated processing of TLB profiles, addressing key bottlenecks and streamlining analysis to support broader adoption of TLB-based diagnostics. The automated baseline-correction algorithm replaces the most time-consuming component of traditional analysis, demonstrating excellent performance for plasma TLB data (characterized by high heat capacity) that was comparable to manual correction. Its performance was less robust for low-signal biofluids, such as urine, where weak thermal features reduce the reliability of baseline estimation. To mitigate this limitation, an ARIMA-based signal-detection algorithm was developed to automatically identify TLB profiles with discernable thermal transitions prior to baseline correction. This pre-screening step effectively filters out noise-dominated data, increasing workflow efficiency, and ensures that downstream analyses focus on interpretable TLB profiles. The signal-detection algorithm achieved near-perfect classification accuracy for TLB profiles with well-defined thermal transitions and maintained a low false-positive rate of 3.1% for true noise profiles, with expected lower performance for borderline cases. Both algorithms have been implemented into the open-source ThermogramForge R Shiny application, providing a unified platform that combines automated processing, expert-guided review, and quantitative TLB profile analysis within a single, user-friendly environment. Future enhancements, including probabilistic signal scoring and adaptive parameter optimization, are expected to further improve scalability and translational potential. Collectively, these developments represent a significant advance toward standardized, efficient, and reproducible TLB-based workflows for both research and clinical applications.

## Figures and Tables

**Table 1:** Performance of the automated signal-detection algorithm compared with expert adjudication.

TLB Profile Signal-to-Noise Ratio	Number of TLB Profiles Evaluated	Raw TLB Data		Baseline-Corrected TLB Data		Reviewer Disagreement (Raw/Baseline-Corrected)
		Correct Classification	Incorrect Classification	Correct Classification	Incorrect Classification	
High	287	286 (99.7%)	1 (0.3%)	285 (99.3%)	2 (0.7%)	1/0
Moderate	97	93 (95.9%)	4 (4.1%)	73 (75.3%)	24 (24.7%)	1/1
Low	172	86 (50%)	86 (50%)	9 (5.2%)	163 (94.8%)	32/7
No Signal	193	154 (79.8%)	39 (20.2%)	187 (96.9%)	6 (3.1%)	13/7

Legend: “Correct classification” and “Incorrect classification” indicates agreement or disagreement with manual adjudication determined by majority rule among three DSC experts. “Reviewer disagreement” shows the number of profiles where a unanimous consensus was not achieved.

## Data Availability

The baseline-correction and signal-detection algorithms are implemented within the *ThermogramBaseline* R package, available through GitHub (http://github.com/BuscagliaR/Thermogram_Baseline). The open-source R Shiny web application ThermogramForge, which provides an integrated workflow for automated analysis of TLB data, is also publicly available through GitHub (https://github.com/BuscagliaR/ThermogramForge) and is hosted free-of-charge through Posit Connect (https://rbuscaglia-thermogramforge.share.connect.posit.cloud/).

## Supplementary Materials

The following supporting information can be downloaded at: https://www.mdpi.com/article/10.3390/cancers18010060/s1, Table S1: TLB profile data for ThermogramForge testing.

## Abbreviations

The following abbreviations are used in this manuscript:

ARIMA	auto-regressive integrated moving average
CSV	comma-separated values
DSC	differential scanning calorimetry
RDS	R data structure
TLB	thermal liquid biopsy
