# Correction: Reger et al. Automated Baseline-Correction and Signal-Detection Algorithms with Web-Based Implementation for Thermal Liquid Biopsy Data Analysis. *Cancers* 2026, *18*, 60

**DOI:** 10.3390/cancers18142222

**Published:** 2026-07-10

**Authors:** Karl C. Reger, Gabriela Schneider, Keegan T. Line, Alagammai Kaliappan, Robert Buscaglia, Nichola C. Garbett

**Affiliations:** 1 Department of Mathematics and Statistics, Northern Arizona University, Flagstaff, AZ 86011, USA; kcr28@nau.edu (K.C.R.); ktl56@nau.edu (K.T.L.); 2 UofL Health—Brown Cancer Center, Louisville, KY 40202, USA; gabriela.schneider@louisville.edu (G.S.); alagammai.kaliappan@louisville.edu (A.K.); 3 Division of Medical Oncology and Hematology, Department of Medicine, University of Louisville, Louisville, KY 40202, USA

## Clarification of Institutional Review Board Statement and Informed Consent Applicability

In the original publication [1], the Institutional Review Board Statement has been updated for clarity. The revised text reads as follows:

**Institutional Review Board Statement:** The study was conducted in accordance with the Declaration of Helsinki. The University of Louisville Institutional Review Board (IRB# 17.0231; reviewed 2 March 2017) determined that the project did not meet the Common Rule definition of human subjects research, as it involved only existing de-identified specimens and data with no ability to link information to individual identities.

Because this study was determined to be non-human subjects research, an Informed Consent Statement was not applicable. For clarity, an Informed Consent Statement has been added to reflect this. The revised text reads as follows:

**Informed Consent Statement:** Not applicable.

The authors state that the scientific conclusions are unaffected. This clarification was approved by the Academic Editor, and the original publication has been updated accordingly.
